# “Short-term effects of a single kangaroo mother care session on urinary allantoin and maternal–infant bonding in preterm neonates: a quasi-randomized controlled trial”

**DOI:** 10.1038/s41598-026-41614-z

**Published:** 2026-03-02

**Authors:** Samreen Manzoor, Samina Kausar, Ayesha Hanif, Faheem Shahzad, Samina Farooqi, Rafia Khalid

**Affiliations:** 1 MS Nursing Scholar, University of Health Sciences, Lahore, Pakistan; 2 Services Hospital & College of Nursing, SIMS/SHL, Lahore, Pakistan; 3 Professor & Head of Nursing Department, University of Health Sciences, Lahore, Pakistan; 4 Consultant Paediatrician, Services Hospital, Lahore, Pakistan; 5 Assistant Professor, Institute of Allied Health Sciences, University of Health Sciences, Lahore, Pakistan; 6 Assistant Professor / Visiting Faculty, Institute of Nursing, University of Health Sciences, Lahore, Pakistan; 7 MPhil Immunology Scholar, University of Health Sciences, Lahore, Pakistan

**Keywords:** Kangaroo mother care, Urinary allantoin, Oxidative stress, Maternal–infant bonding, Preterm neonates, Health care, Medical research

## Abstract

Preterm birth is a leading contributor to neonatal morbidity and mortality, often necessitating neonatal intensive care unit (NICU) admission. The resulting separation of mother-infant dyads may escalate physiological stress and impair early bonding. While Kangaroo Mother Care (KMC) is a well-established intervention, evidence regarding the immediate physiological and affective impact of an isolated, short-duration session remains sparse. Thus, this study aimed to evaluate the short-term effects of a single one-hour KMC session on oxidative stress and maternal–infant bonding in preterm neonates. This quasi-randomized controlled trial was conducted at a tertiary care facility in Lahore, Pakistan (February–July 2024). Forty preterm neonate–mother dyads were allocated based on medical record numbers to receive either a single 60 minute KMC session (*n* = 20) or standard incubator care (*n* = 20). Primary and secondary outcomes included urinary allantoin (measured via ELISA) and Mother–Infant Bonding Scale (MIBS) scores, respectively, assessed at baseline and one hour after the intervention. Data were analysed using paired t-tests and analysis of covariance (ANCOVA). Baseline groups were comparable (p>0.05). Post-intervention, KMC significantly reduced allantoin levels compared to controls (Adjusted Mean Difference [AMD]: −26.18 µmol/mmol; 95% CI: -36.88 to −15.48;* p*<0.001; partial η^2^ =0.399). MIBS scores also improved significantly in the KMC group (AMD: −13.99; 95% CI: −14.84 to −13.14;* p*<0.001; partial η^2^ =0.967). A single one-hour session of Kangaroo Mother Care is associated with reduction in oxidative stress biomarkers and a significant short-term improvement in maternal–infant bonding. These findings suggest that even brief sessions of skin-to-skin contact may serve as potentially effective acute physiological and psychological stabilizer in the NICU setting. However, these immediate effects should be viewed as acute triggers rather than definitive markers of long-term clinical or developmental outcomes.

*Trial registration*: ClinicalTrials.gov, NCT 06338410. Registered 29 March 2024, https://clinicaltrials.gov/study/NCT06338410.

## Introduction

Preterm birth, defined as birth before the completion of 37 weeks of gestation^1^, remains a critical global public health challenge, contributing substantially to neonatal morbidity and mortality. Worldwide prevalence ranges from approximately 9.3% to 12.6%, with some of the highest burdens reported in South Asia, including Pakistan and Bangladesh^2^. In Pakistan, prematurity-related complications account for a high proportion of neonatal deaths, within a context where infant mortality rates reach 62 per 1,000 live births in rural regions^3–5^. Preterm neonates frequently require prolonged admission to neonatal intensive care units (NICUs), imposing substantial emotional, social, and financial burdens on families and healthcare systems^6,7^.

Beyond physical morbidity, NICU hospitalization necessitates early mother–infant separation, which may disrupt the establishment of maternal–infant bonding—the foundational emotional attachment process initiated shortly after birth. This disruption is associated with increased maternal distress, including guilt, grief, and helplessness, and elevates the risk of postpartum depression and post-traumatic stress symptoms^8,9^. In Pakistan, where postnatal depression affects approximately 37% of mothers, there is a clear clinical imperative for interventions that facilitate early maternal–infant interaction^3^.

Physiologically, preterm neonates are uniquely vulnerable to oxidative stress, characterized by an imbalance between the production of reactive oxygen species (ROS) and immature antioxidant defences^10^. This imbalance is often exacerbated by NICU-specific stressors, such as supplemental oxygen, mechanical ventilation, and procedural handling^11,12^.

Oxidative stress is implicated in the pathogenesis of several severe neonatal sequelae, including bronchopulmonary dysplasia, necrotizing enterocolitis, and impaired neurodevelopment^13^. Urinary allantoin, a non-invasive purine oxidation product, has emerged as a reliable biomarker for assessing acute oxidative stress in neonatal populations when normalized to creatinine levels^14,15^.

Kangaroo Mother Care (KMC), a family-centered intervention involving skin-to-skin contact, has been endorsed by the World Health Organization (WHO) and UNICEF to improve thermoregulation, cardiorespiratory stability, and breastfeeding outcomes^16–19^. While current guidelines advocate for prolonged KMC (8–24 h per day) to reduce mortality and infection risk^20,21^ emerging evidence suggests that even shorter sessions may elicit acute physiological and psychological benefits, including reductions in stress biomarkers and improved maternal affective responses^22–25^.

Several studies suggest that KMC may modulate oxidative stress pathways. For instance, reductions in creatinine-normalized urinary allantoin levels have been observed following brief periods of skin-to-skin contact, suggesting a rapid physiological stabilization in response to maternal proximity^14^. Similarly, maternal–infant bonding scores and mood indicators have been reported to improve following repeated KMC exposure^23–28^. However, most existing literature focuses on the cumulative effects of prolonged or repeated sessions. Evidence regarding the acute, short-term responses to a single standardized KMC session remains limited, particularly within the socio-cultural and clinical context of low- and middle-income countries (LMICs).

Despite the established benefits of KMC, data from Pakistan remain scarce, and few studies have simultaneously integrated physiological stress biomarkers with maternal–infant bonding assessments. Furthermore, the immediate physiological and affective “trigger” effects of a single session have not been well-characterized. Therefore, this quasi-randomized controlled trial aimed to evaluate the short-term association between a single 60-minute Kangaroo Mother Care session and changes in urinary allantoin levels and maternal–infant bonding scores in preterm neonates and their mothers at a tertiary care facility in Lahore, Pakistan.

## Methodology

### Study design and setting

This Parallel-arm quasi-randomized controlled trial was conducted in the Neonatal Intensive Care Unit (NICU) of Services Hospital, a tertiary care facility in Lahore, Pakistan, in collaboration with the University of Health Sciences (UHS). Data collection took place between February and July 2024. The study protocol was approved by the Institutional Review Board of Services Hospital (IRB No: IRB/2023/1130/SIMS) and adhered to the Declaration of Helsinki and registered on with trial registration number ID NCT06338410 on ClinicalTrials.gov (available at https://clinicaltrials.gov/*).* No changes were made to the study protocol or outcomes after registration.

### Participants and eligibility

Preterm neonate–mother dyads were eligible if the infants met the following criteria: gestational age of 32–36 weeks, birth weight of 1,500–2,499 g, and a Score for Neonatal Acute Physiology with Perinatal Extension-II (SNAPPE-II) < 9, indicating medical stability. Mothers were included if they were medically stable and provided written informed consent. Exclusion criteria for neonates included major congenital anomalies, grade 3 or higher intraventricular haemorrhage, recent surgery, severe cyanotic heart disease, or the use of sedative/analgesic medications (e.g., morphine, fentanyl, and midazolam). Mothers with acute postpartum complications (e.g., haemorrhage, severe illness) were excluded.

### Sample size and power

Based on effect sizes for urinary allantoin reported by Forde et al., a sample of 20 dyads per group was estimated to provide 80% power at alpha = 0.05 to detect a clinically meaningful difference in oxidative stress markers between the KMC and standard care groups^14^. The total sample of 40 dyads was deemed sufficient to account for the acute nature of the intervention.

### Allocation and blinding

Forty (*n* = 40) dyads were assigned to either the KMC or standard care group using a quasi-randomization technique. Allocation was performed based on the terminal digit of the infant’s medical record number from a fully centralized, hospital-wide automated electronic system that are computer-generated medical record number (odd numbers for KMC, even numbers for control). We explicitly acknowledge that this terminal-digit allocation represents a quasi-randomization approach and may be potentially predictable, posing a risk of selection bias. To mitigate this, allocation was implemented by the Unit In-Charge, who was not involved in participant recruitment, intervention delivery, or outcome assessment. While mothers could not be blinded to the intervention, blinding was strictly maintained for the laboratory staff performing ELISA assays, the outcome assessors for biochemical data, and the data analysts. Samples were de-identified and coded to ensure objective measurement.

### Intervention protocol

The primary investigator and attending physician in Kangaroo Mother Care ward adjacent to NICU helped mothers in providing KMC to their premature infants ( Father and other health care providers were not involved in providing KMC to Premature infants). KMC group received a single 60-minute Kangaroo Mother Care session on the third day of life, in addition to routine care. Following a 15-minute standardized orientation on positioning, infants were placed in skin-to-skin contact on the mother’s chest in a specialized KMC chair. Neonates were continuously monitored via pulse oximetry and temperature probe. The control group received conventional incubator care only. No adverse events (e.g., desaturation, hypothermia and clinical distress like excessive crying) were observed during the study period. Data collection takes place one hour before and one hour after the single one hour session of KMC. This one-hour post-intervention time point was calculated from the end of the skin-to-skin contact. To ensure internal validity, urinary allantoin samples and MIBS questionnaires were collected concurrently from the dyads following the session. This timing was chosen to capture the peak acute physiological and affective response to the stimulus.

### Outcome measures

#### Primary outcome: urinary allantoin 

Urine was collected non-invasively using adhesive collection bags immediately before and one hour after the intervention. Samples were transported by placing in ice box, centrifuged (3,000 rmp for 15 min), and stored at − 80 °C. Urinary allantoin was measured using a commercially available ELISA kit (Shanghai Ideal Medical Technology Co., China) according to the manufacturer’s instructions. Results were normalized to urinary creatinine (µmol/mmol) to adjust for neonatal urine concentration.

####  Secondary outcome: maternal–infant bonding

Bonding was assessed using the Mother–Infant Bonding Scale (MIBS)^29^, where scores range from 0 to 24 (higher scores indicate poorer bonding). The tool was culturally adapted via forward/backward translation (CVI = 0.95, Cronbach’s alpha = 0.78). As the MIBS was administered twice within 24 h to capture acute affective shifts, the potential for recall bias is acknowledged as a limitation.

### Statistical analysis

All statistical analyses were performed using IBM SPSS Statistics version 27.0. Descriptive statistics were utilized to summarize the data; continuous variables, including gestational age, birth weight, urinary allantoin levels, and MIBS scores, are presented as mean ± standard deviation (SD). Categorical variables, such as infant gender, mode of delivery, maternal education level, and socioeconomic status, are reported as frequencies and percentages.

Data were analysed using a hierarchical strategy to enhance transparency and statistical rigor. Baseline characteristics were compared between the Kangaroo Mother Care (KMC) and standard care groups using independent-samples t-tests for continuous variables and chi-square tests for categorical variables.

Post-intervention outcomes were evaluated using a dual-model framework. First, unadjusted between-group differences in post-intervention scores were examined using independent-samples t-tests. Second, the primary adjusted analysis was conducted using analysis of covariance (ANCOVA) to control for baseline variability and improve precision of the treatment effect. In these models, post-intervention outcomes (allantoin levels and MIBS scores) were specified as dependent variables, group allocation as the fixed factor, and the corresponding baseline values were included as covariates.

From the ANCOVA models, estimated marginal means (adjusted means) and adjusted mean differences were obtained with corresponding 95% confidence intervals (CIs). The magnitude of the intervention effect was quantified using partial eta squared (η²_p_). Based on Cohen’s conventions, η²_p_ values of 0.01, 0.06, and 0.14 were interpreted as small, medium, and large effect sizes, respectively.

Within-group changes from baseline to post-intervention were assessed using paired-samples t-tests and *p* < 0.05 was considered statistically significant.

### Ethical considerations

The study protocol was approved by the Ethical Review Committee of the University of Health Sciences, Lahore (Ref. No: UHS/Education/126 − 23/6323k) and the Institutional Review Board of Services Institute of Medical Sciences (SIMS)/Services Hospital (Ref. No: IRB/2023/1130/SIMS).

All study procedures were conducted in strict accordance with the ethical principles outlined in the Declaration of Helsinki. Prior to enrollment, the study’s objectives, procedures, and potential benefits were explained to the participating mothers in their local language. Written informed consent was obtained from all mothers for their own participation and on behalf of their neonates. Participants were assured of their right to withdraw from the study at any time without penalty or impact on their clinical care. Confidentiality and data privacy were maintained throughout the study using de-identified coding systems. The 60-minute Kangaroo Mother Care session was conducted under continuous clinical monitoring to ensure the safety and comfort of the mother–infant dyad.

## Results

**Fig. 1:**
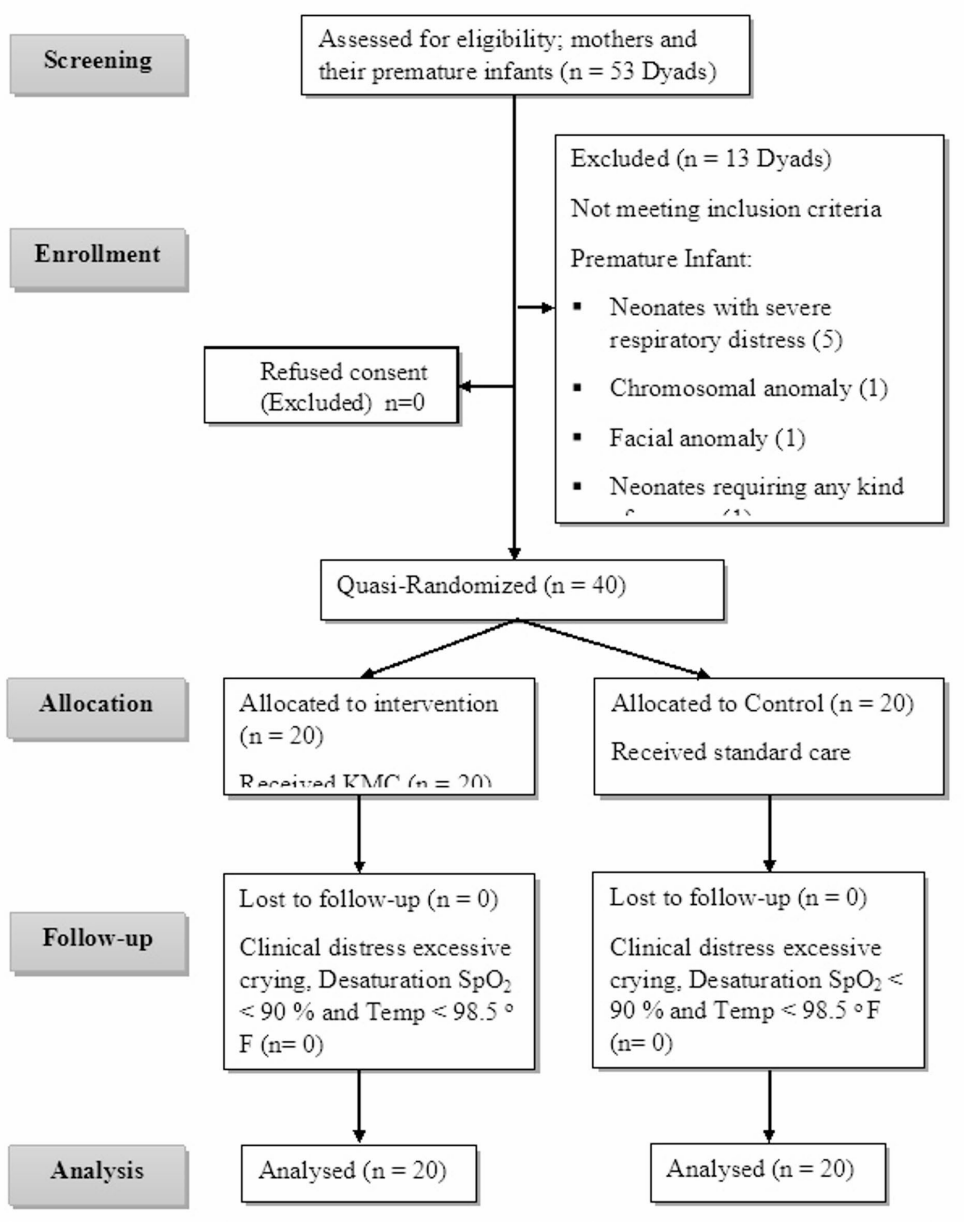
CONSORT flow diagram showing the screening, quasi-randomization, and analysis of mother-preterm neonate dyads. A total of 53 dyads were screened, and 40 were randomized into the Kangaroo Mother Care (*n* = 20) and Standard Care (*n* = 20) groups. All quasi-randomized participants completed the study and were included in the final analysis.

A total of 53 premature neonate–mother dyads were screened for eligibility, of which 13 were excluded for not meeting the inclusion criteria. The remaining 40 dyads were randomized into the KMC (*n* = 20) or standard care (*n* = 20) groups. All participants completed the protocol and were included in the final analysis (Fig. 1).

The data of each control and intervention group is elaborated in three sections:

*Section I* Demographic Variables of Premature Infants and their Mothers

*Section II* Data about Selected Biomarker of Oxidative Stress (Allantoin)

*Section III* Data about Mother-Infant Bonding Scale

### Demographic and baseline characteristics

Baseline demographic characteristics of the preterm infants and their mothers are presented in Table 1. Randomization resulted in two comparable groups, with no statistically significant differences observed regarding gestational age, birth weight, gender distribution, maternal age, mode of delivery, or socioeconomic status (all *p* > 0.05). These findings confirm the baseline homogeneity of the study population.

**Table 1:** Comparison of the demographic characteristics of the participants between the control and intervention groups.

Variable	Control (*n* = 20)	KMC (*n* = 20)	*p*-value
Infant Characteristics			
Gestational age (weeks), Mean ± SD	33.95 ± 0.99	33.75 ± 1.16	0.650*
Gender (Male/Female), n (%)	10 (50%) / 10 (50%)	9 (45%) / 11 (55%)	0.752 ^
Weight (kg), Mean ± SD	1.825 ± 0.20	1.92 ± 0.30	0.251*
*Maternal Characteristics*			
Age (years), n (%)			0.320 ^
20–25	12 (60%)	8 (40%)	
26–30	4 (20%)	7 (35%)	
31–40	4 (20%)	5 (25%)	
Mode of delivery (CS/SVD), n (%)	8 (40%) / 12 (60%)	6 (30%) / 14 (70%)	0.507 ^
Socioeconomic status, n (%)			0.224 ^
Lower / Middle / Upper	5 (25%)/11 (55%)/4 (20%)	9 (45%)/10 (50%)/1 (5%)	
Maternal Education, n (%)			0.841 ^
Primary or less	4 (20%)	5 (25%)	
Secondary	10 (50%)	9 (45%)	
Higher Education	6 (30%)	6 (30%)	

Continuous variables are presented as Mean ± SD and compared using Independent T-tests*. Categorical variables are presented as frequencies (%) and compared using Chi-square χ^2^ tests ^. *p* < 0.05 is considered statistically significant.

#### Urinary biomarkers of oxidative stress (allantoin)

Baseline urinary allantoin levels (normalized to creatinine) were comparable between the standard care group (63.15 ± 5.98 µmol/mmol) and the Kangaroo Mother Care (KMC) group (57.25 ± 12.30 µmol/mmol), with no statistically significant difference observed (t = 1.91, *p* = 0.06), indicating similar baseline oxidative stress status (Table 2).

**Table 2:** Urinary Allantoin Levels (µmol/mmol) before and After KMC (ANCOVA-adjusted).

Variable	Control (*n* = 20)	KMC (*n* = 20)	Statistics & Effect Size
Baseline Mean (± SD)	63.15 ± 5.98	57.26 ± 12.30	t = 1.91, *p* = 0.061
Adjusted Post-Mean (± SE)	66.29 ± 3.65	40.11 ± 3.65	–
Adjusted Mean Difference	26.18 (95% CI: 15.48 to 36.88)		F = 24.58, *p* < 0.001 (ANCOVA)
Mean Change (Δ)	+ 3.60	−17.60	t = 6.89, *p* < 0.001
Within-Group Paired t-test	t(19) = 2.44, *p* = 0.025	t(19) = −3.26, *p* = 0.004	–
Effect Size	–	–	Partial η2 = 0.40; d = 1.20

(Note: Adjusted Post-Means and Adjusted Mean Differences are derived from ANCOVA models with baseline scores as a covariate. )Following the intervention, adjusted post-intervention comparisons demonstrated substantially lower allantoin levels in the KMC group compared with the standard care group. Analysis of covariance (ANCOVA), controlling for baseline values, revealed a statistically significant effect of group allocation on post-intervention allantoin levels [F^1,37^= 24.58, *p* < 0.001, partial η^2^ = 0.399], indicating a large treatment effect. The adjusted mean allantoin level was 40.11 ± 3.65 µmol/mmol in the KMC group and 66.29 ± 3.65 µmol/mmol in the standard care group, yielding an adjusted mean difference of 26.18 µmol/mmol (95% CI: 15.48–36.88).

Within-group analyses showed no statistically significant change from baseline in the standard care group (t^19^= − 1.99, *p* = 0.057), whereas the KMC group demonstrated a significant reduction in allantoin levels following the intervention (t^19^= 6.88, *p* < 0.001). The between-group effect size was large (Cohen’s d = 2.23).

#### Maternal-infant bonding (MIBS scores)

Baseline maternal-infant bonding scores were comparable between the standard care group (19.10 ± 1.37) and the Kangaroo Mother Care (KMC) group (19.20 ± 1.44), with no statistically significant difference observed (t = 0.23, *p* = 0.823), indicating equivalent levels of initial bonding impairment across both groups (Table 3).

**Table 3:** Maternal-Infant Bonding Scale (MIBS) Scores Before and After KMC (ANCOVA-adjusted).

Variable	Control (*n* = 20)	KMC (*n* = 20)	Statistics & Effect Size
Baseline Mean (± SD)	19.10 ± 1.37	19.20 ± 1.44	t = 0.23, *p* = 0.823
Adjusted Post-Mean (± SE)	16.12 ± 0.30	2.13 ± 0.30	–
Adjusted Mean Difference		13.99 (95% CI: 13.14 to 14.84)	F = 1085.64, *p* < 0.001 (ANCOVA)
Mean Change (Δ)	-3.00	−17.05	t = 27.26, *p* < 0.001
Within-Group Paired t-test	t(19) = -8.62, *p* < 0.001	t(19) = −44.84, *p* < 0.001	–
Effect Size	–	–	Partial η^2^ = 0.97; d = 8.62

Note: Adjusted Post-Means and Adjusted Mean Differences are derived from ANCOVA models with baseline scores as a covariate.Following the intervention, adjusted post-intervention comparisons demonstrated substantially lower MIBS scores (indicating improved bonding) in the KMC group compared with the standard care group. Analysis of covariance (ANCOVA), controlling for baseline values, revealed a statistically significant effect on post-intervention bonding levels [F^1,37^= 1085.64, *p* < 0.001, partial η² = 0.967], representing an extremely large treatment effect. The adjusted mean MIBS score was 2.13 ± 0.30 in the KMC group compared to 16.12 ± 0.30 in the standard care group, yielding an adjusted mean difference of 13.99 points (95% CI for adjusted means: KMC [1.53–2.74]; Control [15.51–16.72]).

Within-group analyses showed that while the standard care group experienced a significant but modest reduction in bonding impairment from baseline (t^19^= -8.62, *p* < 0.001), the KMC group demonstrated a profoundly greater clinical reduction (t^19^= -44.84, *p* < 0.001). The magnitude of the difference in change between groups was substantial, as evidenced by a large between-group effect size (Cohen’s d = 8.62).

**Fig. 2:**
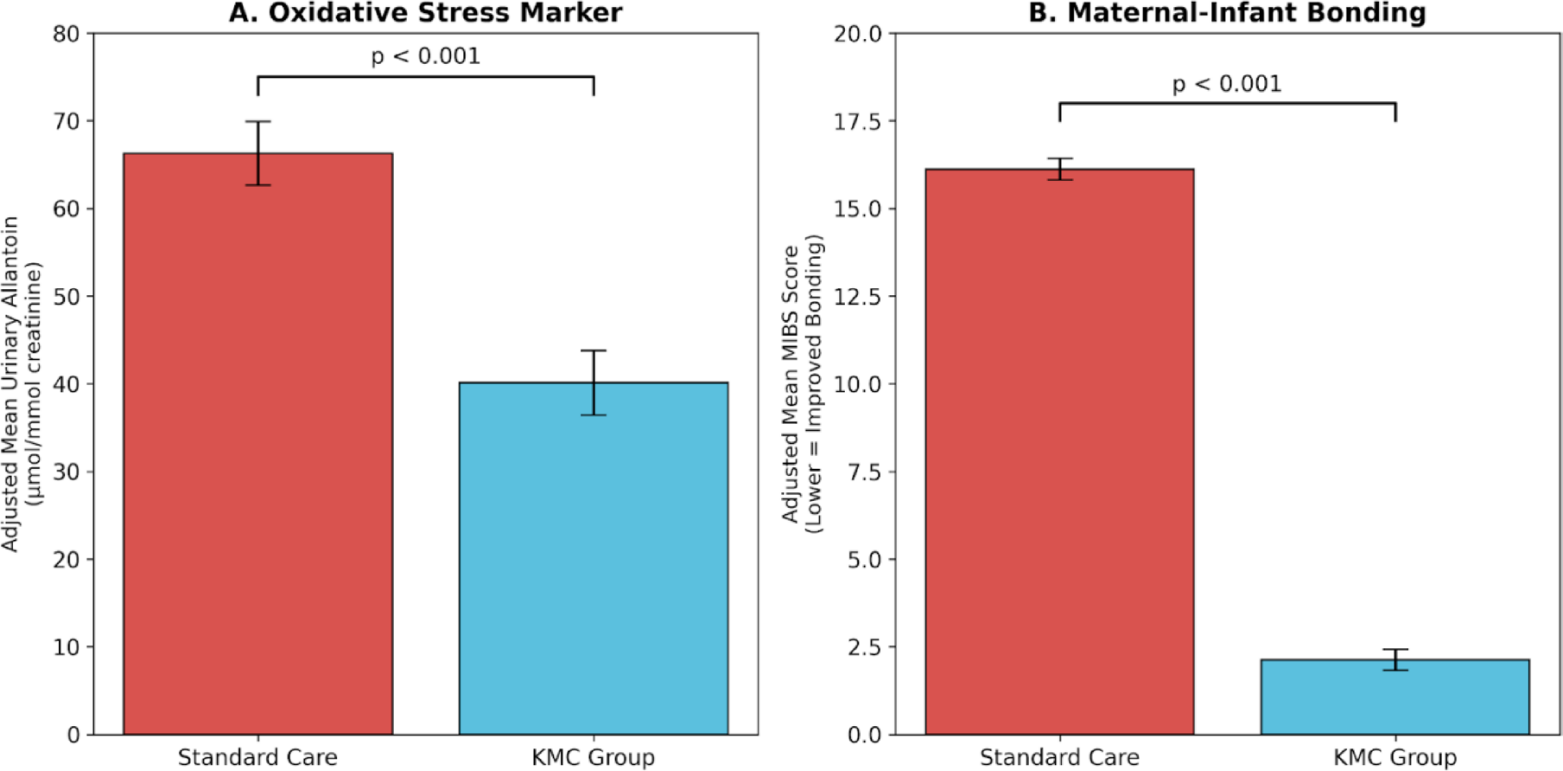
Comparison of adjusted post-intervention outcomes between groups. (**A**) Adjusted mean urinary allantoin levels (µmol/mmol creatinine) showing a significant reduction in oxidative stress markers in the KMC group compared to standard care. (**B**) Adjusted mean Mother–Infant Bonding Scale (MIBS) scores, where lower scores indicate a significant acute improvement in maternal affective bonding. All values are presented as Estimated Marginal Means ± Standard Error (SE) from the ANCOVA model, adjusted for baseline levels. *P* < 0.001 for both outcomes.

The significant reduction in oxidative stress and improvement in bonding following the KMC intervention is visualized in Fig. 2. Panel A illustrates the reduction in adjusted mean urinary allantoin levels in the KMC group compared to standard care (40.11 vs. 66.29 µmol/mmol). Panel B demonstrates the substantial drop in adjusted MIBS scores (2.13 vs. 16.12), representing the immediate affective bonding response.

## Discussion

This quasi-randomized controlled trial investigated the short-term effects of a single session of Kangaroo Mother Care (KMC) on oxidative stress and maternal–infant bonding in preterm neonates. Our findings demonstrate that a 60-minute KMC session on the third day of postnatal life is associated with significant short-term improvements in both biological and psychosocial markers.

The choice of a single 60-minute KMC session as the exposure window is supported by the biological understanding of ‘acute-phase’ skin-to-skin contact. Prior research indicates that maternal-infant proximity can rapidly modulate the neuroendocrine system, specifically through the pulsatile release of oxytocin and the suppression of the hypothalamic-pituitary-adrenal (HPA) axis within 30 to 60 min. These shifts are capable of exerting immediate antioxidant effects and altering maternal affective states^14^.

While prolonged KMC (8–24 h/day) is the gold standard for long-term growth and survival, this initial trial aimed to isolate the immediate stabilization potential of KMC. We did not extend follow-up in this pilot phase to minimize confounding variables such as nursing handovers, environmental noise, and varied feeding schedules that occur over longer durations.

### Effect of KMC on oxidative stress

At baseline, urinary allantoin levels—normalized to creatinine—showed no statistically significant difference between the Kangaroo Mother Care (KMC) and conventional care groups. This equilibrium at the pre-intervention stage confirms the homogeneity of the study population and indicates that both groups were subject to comparable levels of oxidative stress under routine incubator care. These observations are consistent with the findings of Forde, Deming^14^ In the United States, who similarly reported no significant inter-group differences in allantoin levels prior to the initiation of KMC.

Following the 60-minute KMC session on the third day of life, a significant reduction in urinary allantoin levels was observed in the intervention group compared to those receiving standard incubator care. This decrease remained significant after adjusting for baseline values using ANCOVA (*p* < 0.001), with a large effect size (partial η² = 0.399). The marked reduction in this biomarker underscores the physiological effectiveness of skin-to-skin contact in mitigating the accumulation of reactive oxygen species (ROS) in preterm neonates. These results support the hypothesis that KMC exerts a protective effect against oxidative stress. Our findings align with Forde, Deming^14^, who also demonstrated a statistically significant difference in allantoin levels between KMC and incubator-care cohorts^14,30^.

Furthermore, research by El-Farrash, Shinkar^31^ in Egypt corroborates these findings, suggesting that parental skin-to-skin contact serves as a potent biological modulator that reduces overall stress levels in premature infants. In contrast, Sarapuk and Pavlyshyn^32^ reported no significant changes in salivary cortisol levels following skin-to-skin contact in a Ukrainian cohort. However, this discrepancy may be attributed to the different physiological pathways measured; while cortisol reflects the activation of the hypothalamic-pituitary-adrenal (HPA) axis, allantoin provides a direct biochemical reflection of oxidative molecular damage. Thus, urinary allantoin may serve as a more sensitive indicator of the immediate physiological stabilization induced by maternal proximity in the NICU setting.

### Effect of KMC on maternal–infant bonding

At baseline, the assessment of maternal–infant bonding revealed no significant differences between the Kangaroo Mother Care (KMC) and conventional care groups across the various items of the Mother–Infant Bonding Scale (MIBS). Both groups exhibited high mean scores, indicative of poor initial bonding experiences—a finding consistent with the well-documented psychological disruption caused by neonatal intensive care unit (NICU) admission and physical separation. These results align with those of Mörelius, Elander^33^, who similarly observed that mothers of preterm infants reported poor bonding experiences and no statistically significant inter-group differences prior to skin-to-skin contact.

Following the one-hour KMC intervention, the results demonstrated a significant difference in mean MIBS scores between the groups (*p* < 0.001). Even after adjusting for baseline scores using ANCOVA, the effect of KMC remained profound (partial η² = 0.967). These findings suggest that immediate skin-to-skin contact acts as a critical “emotional bridge,” facilitating a rapid reconnection between mothers and their premature infants. Mothers in the intervention group experienced a substantial reduction in bonding impairment scores, indicating a stronger acute affective bond compared to those in the conventional care group, whose infants remained in incubator care. These results are consistent with the work of El-ayari, Mostafa^19^, who reported that KMC enhances maternal bonding behaviors, such as gazing and smiling.

Similarly, Hassan and Shaker^34^ demonstrated that mothers engaging in KMC exhibited significantly higher levels of maternal sensitivity and emotional connection. The observed benefits extend beyond bonding; early skin-to-skin contact has been associated with successful breastfeeding initiation and reduced maternal anxiety^19,35,36^. Furthermore, qualitative evidence suggests that KMC mitigates the “psychological distance” created by the NICU environment, allowing mothers to become familiar with their infant’s health status and fostering a sense of calm^37^.

The potential for KMC to reduce the risk of postpartum depression and bonding failure has been highlighted by Mehler, Hucklenbruch-Rother^22^. Conversely, Forde, Boskovic^38^ reported a worsening of bonding scores following KMC in a United States-based cohort. This discrepancy may be attributed to differences in maternal ethnicity or the wide cultural variations among participants in their study. In contrast, the current study’s findings suggest that in the specific clinical and cultural context of Pakistan, a single session of KMC provides a potent and immediate stimulus for improving the short-term maternal–infant relationship. The observed reduction in MIBS scores within the KMC group—moving from a mean of ~ 19 to ~ 2 within a single hour—represents an exceptionally large effect size (partial η² = 0.967). While this suggests a significant acute affective shift, this magnitude likely reflects several contributing factors beyond the physical intervention itself. Specifically, the immediate post-skin-to-skin period may induce ‘emotional priming,’ where mothers are more predisposed to report positive feelings. Furthermore, the lack of participant blinding may have introduced social desirability bias, where mothers report improved bonding in alignment with cultural expectations of KMC. Additionally, the short one-hour interval between repeated administrations of the MIBS could lead to measurement reactivity. Consequently, these findings should be interpreted as an assessment of immediate affective state rather than the formation of an enduring bond. Further research with longitudinal follow-up and objective, observation-based tools is required to confirm the durability of this effect.”

### Clinical and nursing implications

As the first nursing-led quasi-randomized trial in Pakistan to utilize non-invasive biochemical markers to evaluate KMC, this study underscores the feasibility of integrating brief skin-to-skin sessions into busy NICU protocols. The results suggest that even limited KMC exposure can serve as a physiological trigger for stability. For nursing practice, these findings advocate for the role of the nurse as a facilitator of early neurodevelopmental care, moving beyond purely technical management to include family-centered interventions that improve both infant and maternal outcomes.

### Strengths and limitations

The primary strength of this study lies in its randomized design and the use of an objective, non-invasive biochemical marker (allantoin). The use of ANCOVA to control for baseline variability further strengthens the internal validity of the findings.

However, several limitations must be acknowledged. The quasi-randomization method based on medical record numbers and the single-center design may limit generalizability. Furthermore, the MIBS was administered twice within 24 h; although it captured an acute emotional shift, it may be subject to a Hawthorne effect or recall bias. Finally, the one-hour duration is significantly shorter than the WHO-recommended 8–24 h, meaning these results reflect acute associations rather than long-term clinical protection against oxidative stress related pathologies.

## Conclusion

A single 60-minute KMC session is associated with a significant reduction in urinary allantoin levels and an immediate improvement in self-reported maternal-infant bonding. While the findings suggest KMC is a potentially effective short-term stabilizing intervention for both physiological stress and maternal affect in preterm neonates, the exceptionally large effect sizes observed in bonding scores necessitate cautious interpretation. While these results support the early integration of KMC into routine care, further longitudinal research is required to determine if these acute physiological and affective changes translate into sustained developmental benefits.

## Data Availability

The datasets used and/or analysed during the current study available from the corresponding author on reasonable request.

## References

[1] De Costa, A. et al. Study protocol for WHO and UNICEF estimates of global, regional, and national preterm birth rates for 2010 to 2019. *PLoS One*. **16** (10), e0258751 (2021). 34669749 10.1371/journal.pone.0258751 PMC8528299

[2] For Maternal, T. A. & Group, N. H. I. A. G. S. Population-based rates, risk factors and consequences of preterm births in South-Asia and sub-Saharan Africa: A multi-country prospective cohort study. *J. Global Health*. **12**, 04011 (2022). 10.7189/jogh.12.04011 PMC8850944 35198148

[3] Ariff, S. et al. Effect of maternal and newborn care service package on perinatal and newborn mortality: A cluster randomized clinical trial. *JAMA Netw. Open.* **7** (2), e2356609–e (2024). 38372998 10.1001/jamanetworkopen.2023.56609 PMC10877450

[4] Swarray-Deen, A. et al. Preterm birth in low-middle income countries.* Best Prac. Res. Clin. Obst. Gynaecol.*** 5**, 102518. (2024). 10.1016/j.bpobgyn.2024.102518 38937155

[5] Hafeez, A. et al. The state of health in Pakistan and its provinces and territories, 1990–2019: A systematic analysis for the Global Burden of Disease Study 2019. *Lancet Global Health*. **11** (2), e229–e43 (2023). 36669807 10.1016/S2214-109X(22)00497-1 PMC10009760

[6] Campanha, P. P. A., MCd, M. B., Prata-Barbosa, A., Rodrigues-Santos, G. & Cunha, A. J. L. A. Exclusive breastfeeding and length of hospital stay in premature infants at a Brazilian reference center for kangaroo mother care. *Jornal de Pediatria*. **100** (4), 392–398 (2024). 38522479 10.1016/j.jped.2024.01.004 PMC11331221

[7] Tamir, T. T. Neonatal mortality rate and determinants among births of mothers at extreme ages of reproductive life in low and middle income countries. *Sci. Rep.* **14** (1), 12596 (2024). 38824152 10.1038/s41598-024-61867-w PMC11144189

[8] Garg, D., Chaudhury, S., Saldanha, D. & Kumar, S. Stress, postpartum depression, and anxiety in mothers of neonates admitted in the NICU: A cross-sectional hospital-based study. *Ind. Psychiatry J.* **32** (1), 48–58 (2023). 10.4103/ipj.ipj_93_22 PMC10236660 37274566

[9] Malouf, R. et al. Prevalence of anxiety and post-traumatic stress (PTS) among the parents of babies admitted to neonatal units: A systematic review and meta-analysis. *EClinicalMedicine* ;**43**. (2022). 10.1016/j.eclinm.2021.101233 PMC8713115 34993425

[10] Matyas, M. et al. The association between maternal stress and human milk concentrations of cortisol and prolactin. *Sci. Rep.* **14** (1), 28115 (2024). 39548101 10.1038/s41598-024-75307-2 PMC11568148

[11] Costa, B., Gouveia, M. J. & Vale, N. Oxidative stress induced by antivirals: Implications for adverse outcomes during pregnancy and in newborns. *Antioxidants* **13** (12), 1518 (2024). 39765846 10.3390/antiox13121518 PMC11727424

[12] Lembo, C., Buonocore, G. & Perrone, S. Oxidative stress in preterm newborns. *Antioxidants* **10** (11), 1672 (2021). 34829543 10.3390/antiox10111672 PMC8614893

[13] Martini, S. et al. Antenatal and postnatal sequelae of oxidative stress in preterm infants: A narrative review targeting pathophysiological mechanisms. *Antioxidants* **12** (2), 422 (2023). 36829980 10.3390/antiox12020422 PMC9952227

[14] Forde, D. et al. Oxidative stress biomarker decreased in preterm neonates treated with kangaroo mother care. *Biol. Res. Nurs.* **22** (2), 188–196 (2020). 31973579 10.1177/1099800419900231 PMC7273802

[15] López-Hernández, Y. et al. The urinary metabolome of newborns with perinatal complications. *Metabolites* **14** (1), 41 (2024). 38248844 10.3390/metabo14010041 PMC10819924

[16] Bergman, N. J. New policies on skin-to-skin contact warrant an oxytocin-based perspective on perinatal health care. *Front. Psychol.* **15**, 1385320 (2024). 39049943 10.3389/fpsyg.2024.1385320 PMC11267429

[17] Organization, W. H. *Kangaroo mother care: A transformative innovation in health care* (World Health Organization, 2023).

[18] Arslan, F. T. et al. Effect of kangaroo mother care on cerebral oxygenation, physiological parameters, and comfort levels in late-premature infants: A randomized controlled trial. *Midwifery* **137**, 104096 (2024). 39024964 10.1016/j.midw.2024.104096

[19] El-ayari, O. S. M., Mostafa, S. S. & El-Salamony, A. A. W. The impact of kangaroo care on psychological bonding, placental separation, and maternal anxiety among primiparas women. *Int. Egypt. J. Nurs. Sci. Res.* **4** (1), 352–369 (2023).

[20] Bisanalli, S. et al. The beneficial effect of early and prolonged kangaroo mother care on long-term neuro‐developmental outcomes in low birth neonates–A cohort study. *Acta Paediatr.* **112** (11), 2400–2407 (2023). 37543716 10.1111/apa.16939

[21] Sivanandan, S. & Sankar, M. J. Kangaroo mother care for preterm or low birth weight infants: A systematic review and meta-analysis. *BMJ Global Health*. **8** (6), e010728 (2023). 37277198 10.1136/bmjgh-2022-010728 PMC10254798

[22] Mehler, K. et al. Delivery room skin‐to‐skin contact for preterm infants—A randomized clinical trial. *Acta Paediatr.* **109** (3), 518–526 (2020). 31423649 10.1111/apa.14975

[23] Mehrpisheh, S. et al. The effectiveness of kangaroo mother care (KMC) on attachment of mothers with premature infants. *Eur. J. Obstet. Gynecol. reproductive biology: X*. **15**, 100149 (2022). 10.1016/j.eurox.2022.100149 PMC9046128 35493996

[24] Cho, H. & Jeong, I. S. The relationship between mother-infant contact time and changes in postpartum depression and mother‐infant attachment among mothers staying at postpartum care centers: An observational study. *Nurs. Health Sci.* **23** (2), 547–555 (2021). 33914405 10.1111/nhs.12847

[25] Kurt, F., Kucukoglu, S., Ozdemir, A. & Ozcan, Z. The effect of kangaroo care on maternal attachment in preterm infants. *Niger. J. Clin. Pract.* **23** (1), 26–32 (2020). 31929203 10.4103/njcp.njcp_143_18

[26] Packheiser, J. et al. A systematic review and multivariate meta-analysis of the physical and mental health benefits of touch interventions. *Nat. Hum. Behav.* :1–20. (2024). 10.1038/s41562-024-01841-8 PMC11199149 38589702

[27] Çağan, E. S. & Genç, R. The effects of kangaroo care at birth on exclusively breastfeeding, baby’s growth and development according to attachment theory: A randomized controlled trial. *Early Child. Dev. Care*. **193** (3), 378–387 (2023).

[28] Cristóbal Cañadas, D., Parrón Carreño, T., Sánchez Borja, C. & Bonillo Perales, A. Benefits of kangaroo mother care on the physiological stress parameters of preterm infants and mothers in neonatal intensive care. *Int. J. Environ. Res. Public Health*. **19** (12), 7183 (2022). 35742429 10.3390/ijerph19127183 PMC9223087

[29] Karakayalı Ay, Ç., Özşahin, Z. & Karataş Okyay, E. The moderating effect of birth satisfaction on the correlation between mother-infant bonding and psychological resilience. (2023).

[30] Forde, D., Fang, M. L. & Miaskowski, C. A systematic review of the effects of skin-to-skin contact on biomarkers of stress in preterm infants and parents. *Adv. Neonatal Care*. **22** (3), 223–230 (2022). 34054011 10.1097/ANC.0000000000000905 PMC9150851

[31] El-Farrash, R. A. et al. Longer duration of kangaroo care improves neurobehavioral performance and feeding in preterm infants: A randomized controlled trial. *Pediatr. Res.* **87** (4), 683–688 (2020). 31493775 10.1038/s41390-019-0558-6

[32] Sarapuk, I. & Pavlyshyn, H. Assessment and correction of stress in preterm infants and their mothers. *Turkish Archives Pediatr.* **57** (2), 146 (2022). 10.5152/TurkArchPediatr.2022.21158 PMC9366232 35383008

[33] Mörelius, E., Elander, A. & Saghamre, E. A Swedish translation and validation of the mother-to-infant bonding scale. *Scand. J. Public Health*. **49** (4), 465–470 (2021). 32156193 10.1177/1403494820910336

[34] Hassan, S. & Shaker, W. Attachment between mother and premature baby: A quasi-experimental study. *J. Curr. Med. Res. Opin.* **7** (04), 2226–2235 (2024).

[35] Cong, S. et al. Skin-to‐skin contact to improve premature mothers’ anxiety and stress state: A meta‐analysis. *Matern. Child Nutr.* **17** (4), e13245 (2021). 34258864 10.1111/mcn.13245 PMC8476413

[36] Wang, Y., Zhao, T., Zhang, Y., Li, S. & Cong, X. Positive effects of kangaroo mother care on long-term breastfeeding rates, growth, and neurodevelopment in preterm infants. *Breastfeed. Med.* **16** (4), 282–291 (2021). 33533688 10.1089/bfm.2020.0358

[37] Føreland, A. M., Engesland, H., Kristoffersen, L. & Fegran, L. Postpartum experiences of early skin-to-skin contact and the traditional separation approach after a very preterm birth: A qualitative study among mothers. *Global Qual. Nurs. Res.* **9**, 23333936221097116 (2022). 10.1177/23333936221097116 PMC9125059 35615558

[38] Forde, D. E. et al. (eds) *The Association of Kangaroo Mother Care, Energy Conservation, Procedural Pain, and Bonding in Preterm Neonates* (28th International Nursing Research Congress, 2017).

